# Inequity in treatment access for child mental health services in England: analysis of administrative national data for 2021–2022

**DOI:** 10.1192/bjb.2024.114

**Published:** 2026-02

**Authors:** Tom Pape, Lauren Rixson, Anees Ahmed Abdul Pari

**Affiliations:** Public Health Directorate (East of England), NHS England, Fulbourn, UK

**Keywords:** Epidemiology, social deprivation, childhood experience, health economics, out-patient treatment

## Abstract

**Aims and method:**

An equitable child mental health service provides access to treatment proportionally to the need of individual demographic groups. Despite qualitative and survey-based evidence of barriers disadvantaging some demographic groups, it is not well understood how these barriers translate into quantifiable inequities. We calculated the treatment access rate for English children aged 6–16 years in 2021–2022, using the patient-level Mental Health Services Data Set and Mental Health of Children and Young People Survey.

**Results:**

The number of primary school children in treatment needs to increase nationally by 173%, the number of boys by 65% and the number of children from a White ethnic background by 31%, to achieve equity in treatment access. There was no evidence of inequities by area deprivation.

**Clinical implications:**

Child mental health services in England should not only increase overall access rates, but also pay more attention to equity in access across different demographic groups.

Poor mental health adversely affects children during a critical period of their development.^1^ Yet, only a third of English children with a diagnosable mental health condition receive treatment.^2^ Explanations for this low treatment access rate include not only a lack of service capacity, but also access barriers ranging from a lack of awareness about service offers and limited ability to recognise mental health problems to negative perceptions of help-seeking by both children and their parents, who often act as ‘gatekeepers’ for children to receive help.^3,4^

Some demographic groups may face additional access barriers. For example, young males’ role expectations can make them hesitant to obtain mental health support.^3,5^ Young ethnic minorities or their parents may delay decisions to seek help based on previous racial mistreatment, language barriers or concerns about family reputation,^6^ which is consistent with their lower self-referral rates.^7^ Younger children, boys and children of Black ethnicity are also more likely to attend only a single appointment, potentially because of a disproportionate need for alternative services or higher drop-out rates.^8^ Although there are surveyed differences in the prevalence of child mental health disorders by demographics,^9^ there is scarce evidence to what extent services match these differences in need in order to be equitable. Therefore, the existence and scale of inequities in treatment access remain unknown. Our paper works toward filling this evidence gap by quantifying the treatment access rate of children aged 6–16 years for England, using a national patient-level data-set and a national prevalence survey.

## Method

We define the treatment access rate as the ratio of the number of children that received treatment (numerator) over the number of children in need of treatment (denominator). In the next two subsections, we describe how we obtained both numbers for English children aged 6–16 years grouped by gender, age, ethnicity and area deprivation, both nationally and for individual National Health Service (NHS) integrated care systems (ICSs), followed by how we measure inequity in treatment access.

Data usage was approved by the NHS England Information Governance team (reference 1356).

### Number of children receiving treatment (numerator)

Our numerator is the number of children aged 6–16 years who received at least two contacts with a mental health professional, with the follow-up contact occurring in 2021–2022. Children are referred to mental health services from various sources, including parents, teachers and primary care physicians. A referral on its own does not necessarily imply that the patient has a disorder requiring treatment. At the first appointment, the mental health professional needs to assess the suitability of the patient for treatment. Some patients may only require a single session for signposting, self-management advice or referral to alternative services. For this reason, only patients with at least two contacts on a referral can be reliably regarded as both requiring and receiving mental health treatment.

This two-contact rule is in line with the traditional NHS definition for the number of patients in treatment in any given year.^10^ As the findings of our study are especially aimed at NHS organisations to develop more equitable child mental health services, we replicate the NHS definition of treatment access rate in our analysis. More precisely, the NHS determines the number of patients in treatment as the number of children who attended their first contact before their 17th birthday (including contacts in previous financial years) and had at least one follow-up contact in the focal financial year (2021–2022 in our case). The patient must have been at least 6 years old at the initial contact. Appointments the patient ‘did not attend’ and SMS/email exchanges are not included as they cannot be considered as receiving treatment. Mental health in-patients are always regarded as being in treatment. We count each patient only once per year, even if they have more than one referral resulting in treatment.

We obtain our count for children receiving treatment from the record-level Mental Health Services Data Set (MHSDS), which is part of the National Commissioning Data Repository held by NHS England. NHS England Information Governance approval was obtained to use the MHSDS for this study. The MHSDS contains data on patient activity with publicly funded mental health services by all major hospitals, community clinics, specialised mental health services and some voluntary sector services.^11^ Our analysis considers the following four data tables in MHSDS:^12^ MHS001 (for patients’ last-recorded NHS number, gender, ethnicity and local super output area to map deprivation), MHS101 (for referral reason and ICS), MHS201 (for out-patient contact dates, age at time of contact and mode of contact) and MHS501 (for in-patient stays and age at time of admission) focusing on the financial year between 1 April 2021 and 31 March 2022. We did not allow for a referral to map onto more than one contact at any point in time, or map onto more than one patient in the MHSDS – both submission issues for several voluntary sector providers. MHSDS includes services for mental, behavioural and neurodevelopmental disorders, with the latter being outside the scope of our study due to different type of service needs. In line with NHS statistics on treatment access,^13^ we exclude all referrals with neurodevelopment disorders as the primary reason (referral codes 24–26 in the MHSDS). As the ethnicity field is frequently recorded as ‘null’ or ‘not stated’ in the MHSDS,^14^ we linked the MHSDS with the in-patient, out-patient and accident and emergency data-sets on the National Commissioning Data Repository to obtain a patient's actual ethnicity. This improved ethnicity recording in our sample from an average of 87 to 97%, in line with recording for other demographics (98% for gender, 95% for local super output area). We were unable to consider the 0.2% of patients in contact with mental health services in 2021–2022 that did not have a recorded age in our analysis. Missing data is assumed to be at random. Our complete SQL extraction code for our analysis can be found in the Supplementary Material available at https://doi.org/10.1192/bjb.2024.114.

### Number of children in need of treatment (denominator)

The denominator is the number of children estimated to have a diagnosable mental health problem in need of treatment. The Mental Health of Children and Young People Survey in England (MHCYP) is used to determine the prevalence of mental health disorders in the population. The MHCYP survey is a national survey with outcomes weighted for representation and non-response.^9^ The survey uses the Strength and Difficulties Questionnaire (SDQ) administered online and via telephone to either the parent or the child. The SDQ responses translate into a prediction of whether a child has a ‘probable’ mental disorder,^15^ resulting in the prevalence measure we use in our analysis.

We use the 2021 MHCYP survey's percentage prevalence estimates for children aged 6–16 years, split by gender, age group and ethnicity (unweighted responses *n* = 2603). The age range 6–16 years is selected because of child mental health services typically serving the population under 18 years old and poor response rate for children aged 17–19 years old within the MHCYP survey. Ethnicity was aggregated to the two groups ‘White’ and ‘Black and minority ethnic’, because of the small sample sizes. We use the 2020 survey (unweighted responses *n* = 2588) to obtain mental disorder prevalence by the Index of Multiple Deprivation, because this is not available in the 2021 survey.

To determine the absolute number of children with a probable mental disorder by these demographic groups, the national MHCYP survey's percentage prevalence estimates are applied to national and ICS-level population counts. We use the Office for National Statistics 2020 mid-year population counts for gender and age,^16^ Index of Multiple Deprivation 2019 quintile^17^ and the estimated subnational 2019 mid-year population counts for ethnicity, as no newer data were available at the time of analysis.^18^

As the population of interest are children with a mental health condition that require treatment (at least two contacts with a professional), the THRIVE framework of child and young person mental health service delivery is used to determine how much of the population in need required this level of support. The THRIVE framework distinguishes services by the needs of different groups of children and young people, rather than by severity or type of problem.^19^ Within this framework, children and young people with milder symptoms only require brief support to normalise behaviour and reassure families through advice or signposting to more appropriate services (‘Getting Advice’ group). The ‘Getting Advice’ group is estimated to make up 28% of the population of children and young people accessing mental health services. The remaining 72% are likely to require treatment (‘Getting Help and Further Help’ groups) beyond a single contact. Therefore, only this population in need of treatment is considered in our analysis, by multiplying all prevalence estimates with a factor of 0.72.

### Calculating inequity in treatment access

Having obtained the numerators and denominators for the treatment access rates, we can compute our pairwise inequity measure for our four sociodemographic characteristics: primary (age 6–10 years) versus secondary school (age 11–16 years), boys versus girls, most versus least deprived quintile and White versus Black and minority ethnic background. Let us denote the treatment access rate for each pair A versus B (e.g. boys versus girls) as *T*_*A*_ and *T*_*B*_, respectively. For simplicity, we assume the treatment access rate of group A is smaller than of group B, so that the absolute difference *T*_*B*_ − *T*_*A*_ is a positive value. To achieve equity in treatment access, an absolute increase of the treatment access rate of group A by *T*_*B*_ − *T*_*A*_ percentage points is required. Or equivalently, this translates into a required relative increase of the treatment access rate of group A by 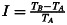 per cent to achieve equity. This relative increase *I* is our measure of inequity in treatment access. For instance, group A may have a treatment access rate *T*_*A*_ of 10% and group B a treatment access rate *T*_*B*_ of 15%, so that we would need to increase the treatment access for group A by *I* = 50% to achieve equity. It is important to note that mental health services cannot change the denominator (i.e. number of patients in need of treatment), only the numerator (number of patients receiving treatment) of the treatment access rate *T*_*A*_. Thus, what we are effectively saying is that the number of treated patients of group A needs to be increased by *I* per cent to achieve equity in access.

As a final technical comment, note that our inequity measure is a ratio, and any proportional changes of both the treatment access rates of groups A and B mathematically cancel out. This especially includes the assumption that only 72% of all patients with a probable mental health disorder need treatment, as the factor 0.72 in the denominators of both *T*_*A*_ and *T*_*B*_ disappear in the ratio. Similarly, an ICS's prevalence rate can be proportionally higher or lower than the national levels across the two groups for each sociodemographic pair (i.e. multiplied across the board by any factor), without this changing our measure for inequity in treatment access *I*.

## Results

Applying our outlined data extraction method on MHSDS, we identified 350 950 English children aged 6–16 years who received mental health treatment in 2021–2022 (see Table 1), of which 56.6% were female, 75.3% were aged 11–16 years, 19.1% were of a Black or minority ethnic background and 26.6% were living in the most deprived quintile. Because of the rapid uptake of telephone consultations, the provision of mental health services in England recovered just a few months into the COVID-19 pandemic. To gain an understanding of the impact of the inclusion criteria on our outcomes, we note that our reported number of children in treatment would have been (a) 42% higher if we had regarded patients with a single appointment as receiving treatment (or mathematically equivalently, just 70% of patients with a first contact proceeded to a follow-up, which is consistent with the THRIVE estimate that 72% of referred patients need help rather than just advice), (b) 21% higher if we had not corrected for duplicate event submissions, (c) 13% higher if we had not excluded patients with a neurodevelopmental disorder as the primary referral reason, (d) 5% higher if we had considered appointments the patient did not attend as contacts (as included in NHS reporting)^13^ and (e) 2% higher if we had counted SMS/email exchanges as contacts.

**Table 1:** Equity outcomes across paired demographics

	All (6–16 years)	Boys	Girls	Primary school (6–10 years)	Secondary school (11–16 years)	White	Black and minority ethnic	Least deprived (IMD quintile 5)	Most deprived (IMD quintile 1)
England population	7 551 626	3 872 074	3 679 552	3 553 355	3 998 271	5 730 118	1 821 508	1 447 803	1 776 096
Patients in treatment^a^ (% of population)	350 950 (4.65%)	145 375 (3.75%)	198 530 (5.40%)	89 215 (2.51%)	264 430 (6.61%)	283 850 (4.95%)	57 160 (3.14%)	50 920 (3.52%)	93 225 (5.25%)
Prevalence of probable mental disorder [95% CI]^b^	17.4% [15.7–19.1]	18.6% [15.9–21.2]	16.2% [14.0–18.4]	17.1% [14.7–19.6]	17.7% [15.4–20.0]	20.1% [18.0–22.1]	9.7% [6.6–12.7]	14.0% [10.8–17.3]	20.8% [16.5–25.0]
Count of population in need of treatment	946 068	518 548	429 183	437 489	509 540	829 264	127 214	145 939	265 988
Treatment access rate^c^	37.1%	28.0%	46.3%	20.4%	51.9%	34.1%	40.4%	34.9%	35.0%
Inequity measure *I* (additional patients needed in treatment to achieve equity)	–	65.0% (+94 492)		154.5% (+137 825)		31.3% (+88 760)		0.5% (+227)	

IMD, Index of Multiple Deprivation.

a. Derived from the Mental Health Services Data Set 2021–2022.

b. Figures from Mental Health of Children and Young People Survey in England 2021 (gender, age, ethnicity) and 2020 (deprivation).

c. As calculated by the prevalence in 2020 Office for National Statistics population estimates and THRIVE framework of children and young people in need of help.

These 350 950 children in treatment equates to 4.65% of the English population of this age group. There are significant differences in the proportion of each population subgroup accessing services, with more girls (by 1.65% points), secondary school children (by 4.1% points), children from a White background (by 1.8% points) and children in the most deprived quintile (by 1.7% points) receiving treatment. However, these crude access outcomes do not consider if this is representative of treatment need.

In 2021, the prevalence of a probable mental disorder in English children aged 6–16 years was 17.4%. Split by demographics, we observe that a larger share of boys (by 2.4% points), secondary school children (by 0.6% points), children in the most deprived quintile (by 6.8% points) and children of White ethnicity (by 10.4% points) were estimated to have a probable mental health disorder.

After relating the number of individuals estimated to need treatment with the number of individuals receiving treatment, we find that services are inequitable. We identify a lower treatment access rate for boys (by 18.3% points), younger children (by 31.5% points) and children from a White background (by 6.3% points). Or put differently, our results indicate that the number of boys in treatment would need to increase by 65%, the number of primary school children by 154% and the number of children from a White ethnic background by 31%, to achieve equity in treatment access. We do not observe inequity in treatment access when comparing the most and least deprived areas, both at 35% of the population in need.

**Fig. 1:**
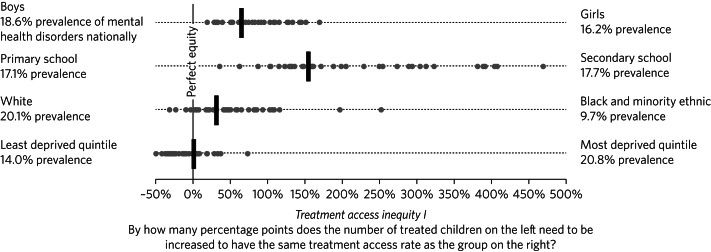
Treatment access inequity measure *I* for 2021–2022, nationally (black bars) and for each of the individual integrated care systems (grey dots).

Figure 1 shows our measure of inequity in treatment access *I* for England as a whole (blue bars), as well as for its 42 ICSs responsible for planning and commissioning most mental health services (grey dots). We see considerable local variation in the level of inequity. Our first observation is that all ICSs in England provide a mental health service that disadvantages boys and primary school children, indicating a fundamental challenge in serving this population group across the country. The picture for ethnicity is more mixed, with six ICSs having a lower treatment access rate for Black and minority ethnic children and 32 ICSs having a higher treatment access rate. There is a relatively even split for deprivation, with 26 ICS offering a slightly higher treatment access rate for the most deprived and 16 ICS for the least deprived quintiles.

## Discussion

In a system with limited resources, child mental health services should prioritise higher-risk demographics to ensure equity. Our results support previous evidence of a treatment gap in English child mental health services,^2^ with only 4.7% of this population in treatment compared with the estimated 17.4% probably experiencing a mental disorder, equating to a treatment access rate of only 37.1%. However, for the first time, we show that this treatment access is also inequitable, underserving boys, younger children and children of White ethnicity.

We demonstrate that the number of boys in treatment would need to increase by 94 492 (or 65%) to achieve the same access rate as girls. Such a gender treatment gap has been previously recognised for mental health in adult men, where gender related attitudes may delay care-seeking.^20^ Although less evidence is available for children and adolescents, similar negative attitudes to help-seeking have been reported in young men.^3^ Such attitudes have been the target of campaigns in Australia that use help-seeking promotion and stigma-reducing interventions to successfully increase service access for men.^21^

We provide evidence that the number of primary school children in treatment would need to increase by 137 825 (or 154%) to match the treatment access rate of secondary school children. There is a lack of research into understanding service access barriers for young children. Consent and recognition of care in this age group are particularly under the control of parents, who may need support in dealing with stigmatising attitudes and understanding treatment needs for young children.^22^

We show that 88 760 (or 31%) more children from a White ethnic background require treatment to reach equity in treatment access with children from Black and minority ethnic backgrounds. This finding may be surprising given the published criticism of mental health service access for ethnic minorities,^6^ and the lower rate of service usage per capita. However, considering the lower prevalence of mental disorders for Black and minority ethnic children, as reported elsewhere,^23,24^ treatment access rates are lower for children from a White background. Children of Black or Asian ethnicity have been found to consult general practice services more frequently than children of White ethnicity, which may increase the chance of a disorder being recognised and referred.^25^ Reports suggest that children from minority ethnic backgrounds are more likely to attend mental health services through compulsory means, which could contribute to higher rates of access compared with those with the choice to self-refer.^7,26^

We do not find evidence of an access inequity by area deprivation, consistent with previous research in adolescents from Sweden.^27^ Research in adults suggests that the contribution of area deprivation to mental health prevalence is likely to be small compared with individual and household factors,^28,29^ but there is generally sparse evidence for children on the impact of deprivation on both prevalence and access.

### Strengths and limitations

This is the first study, to our knowledge, to use existing national mental health data to consider treatment access rates for child mental health services across demographic characteristics and explore healthcare inequity. Our proposed measure can be applied by analysts at a local level to inform the planning efforts of commissioners and providers. We captured access inequity in a single number, which is the kind of information managers tend to be most ready to act on. A strength of our study is the use of the MHSDS, a national administrative data-set, which reduces concerns about sampling biases and small sample sizes. Notably, our 97% ethnicity coverage by linking the MHSDS data with national acute hospital data rules out systematically biased ethnicity recording in our administrative data as an alternative explanation for our findings. By comparing the MHSDS with the MHCYP survey, we bridge the gap between service use and prevalence to assess unmet healthcare needs and highlight systematic differences across demographic groups.

Although we mitigated many data-quality concerns with the MHSDS, there were unresolved missing data issues. Primary referral reason was not recorded for 12% of children, possibly leading to a 2% overestimation of treatment access rates because we could not filter out all referrals for neurodevelopmental disorders, as per our inclusion criteria. For the same reason, we were unable to analyse inequity by type of mental health disorder. Although we corrected for the double submission issue by some voluntary providers in the MHSDS, 9% of treatments could not be linked to a unique NHS number, potentially causing some patients to be counted twice. Furthermore, 118 out of 454 mental health service providers did not consistently submit data to the MHSDS until March 2022;^12^ however, these 118 providers were all small local services, and did not include any NHS mental health trusts, which deliver the majority of treatments.

The MHCYP survey provides a representative estimate of mental health disorders in children and young people across England, including those who may be experiencing mental health problems but have not sought care. However, as is common with surveys of this size, the self-reported data at a single point in time is subject to recall and response bias, which may lead to under- or overreporting. Furthermore, the SDQ, which underpins the MHCYP survey, is primarily a screening tool and not a diagnostic instrument. It has been found to have a low sensitivity (<50%) for certain anxiety and eating disorders, and therefore may not capture the full range of mental health difficulties experienced by children.^15^ Evidence also suggests that parent and child self-completed outcomes may not be aligned, leading to potential biases based on the subjective observations of the responder.^30^ However, for the purpose of a national survey, the SDQ has the advantage of allowing for a standardised outcome across a range of potential diagnoses. It is accessible and can be completed remotely by survey participants, which is essential where a high response rate is necessary for adequate population representation. Additionally, the SDQ has been validated to compare outcomes across different ethnicities and deprivation levels in the UK, reducing concerns about accounting for cultural sensitivity in measures.^31^

We included only patients who had at least two contacts with services, as they are more likely to have a diagnosable mental health disorder requiring professional intervention. However, this may be mismatched to the SDQ, which as a screening tool, can only provide an indication for further clinical evaluation. It is unclear if those with milder symptoms would actively seek or require care in a specialist NHS setting, particularly in areas with access to early intervention services, and how this contributes toward our identified care gaps.^32^ Better recording of referral reasons in the MHSDS to allow for a condition-specific analysis may provide further insights into this issue. We also recognise that MHSDS submissions from private or tertiary services that receive no NHS funding are voluntary, potentially underestimating overall service use.

Although data suggests that mental health services were minimally affected by the COVID-19 pandemic,^13^ our outcomes represent only a single year. This paper does not study if inequities have shifted as a result of pandemic-related changes in service delivery methods and the worsening of mental health disorder prevalence.

Public health research often deals with broader population-level data and trends, which can introduce uncontrollable variability, as previously described. Such variability might not be as pronounced in more focused mental health studies, such as the evolving trends in how distress in young people is perceived, especially in the post-pandemic context. Although our methods provide a valuable overview, they do not capture the full complexity of individual experiences and local variations in service access. Mixed-method primary data collection, although resource-intensive, could offer deeper insights and help validate our findings in specific contexts. However, it is also a reality that decision makers typically do not have the capacity to deploy mixed-method primary data collection to their local areas, and our study provides an alternative approach.

In conclusion, access to mental health services for children and young people in England varies by gender, age and ethnicity, and these differences are not equal to need. This inequity places certain demographics at a higher risk of developing mental health problems if they cannot gain sufficient access to treatment early in life, potentially increasing the demand for unscheduled care. Documenting these access inequalities is complex, with different methodologies contributing to the emerging discussions and evidence base on this topic. The purpose of this paper was to explore what can be learned from existing national data about equity in access to child mental health services, alongside considering the strengths and limitations of this approach.

## Supporting information

Pape et al. supplementary material Pape et al. supplementary material

## Data Availability

Access to the administrative patient-level data used in this study can be obtained through NHS England's Data Access Request Service (DARS).

## About the authors

**Tom Pape** and **Lauren Rixson** are healthcare public health officers at the Public Health Directorate (East of England), NHS England, Fulbourn, UK. **Anees Ahmed Abdul Pari** is Deputy Managing Director of the Strategic Commissioning Unit for the Cambridgeshire and Peterborough Integrated Care Board, Ely, UK.
